# Full-color generation enabled by refractory plasmonic crystals

**DOI:** 10.1515/nanoph-2022-0071

**Published:** 2022-05-06

**Authors:** Zong-Yi Chiao, Yu-Chia Chen, Jia-Wern Chen, Yu-Cheng Chu, Jing-Wei Yang, Tzu-Yu Peng, Wei-Ren Syong, Ho Wai Howard Lee, Shi-Wei Chu, Yu-Jung Lu

**Affiliations:** Research Center for Applied Sciences, Academia Sinica, Taipei 11529, Taiwan; Department of Physics, National Taiwan University, Taipei 10617, Taiwan; Department of Physics & Astronomy, University of California, Irvine, CA 92697, USA; Molecular Imaging Center, National Taiwan University, Taipei 10617, Taiwan; Brain Research Center, National Tsing Hua University, Hsinchu 300044, Taiwan

**Keywords:** HfN, localized surface plasmon resonance, plasmonic colors, plasmonic crystals, refractory plasmonics, transition metal nitrides

## Abstract

Plasmonic structural color, in which vivid colors are generated via resonant nanostructures made of common plasmonic materials, such as noble metals have fueled worldwide interest in backlight-free displays. However, plasmonic colors that were withstanding ultrahigh temperatures without damage remain an unmet challenge due to the low melting point of noble metals. Here, we report the refractory hafnium nitride (HfN) plasmonic crystals that can generate full-visible color with a high image resolution of ∼63,500 dpi while withstanding a high temperature (900 °C). Plasmonic colors that reflect visible light could be attributed to the unique features in plasmonic HfN, a high bulk plasmon frequency of 3.1 eV, whichcould support localized surface plasmon resonance (LSPR) in the visible range. By tuning the wavelength of the LSPR, the reflective optical response can be controlled to generate the colors from blue to red across a wide gamut. The novel refractory plasmonic colors pave the way for emerging applications ranging from reflective displays to solar energy harvesting systems.

## 1 Introduction

The engineering of structural colors is a promising and rapidly emerging research field that could have a significant technological impact on the backlight-free display market. Eco-friendly structural color without a specific pigment is a phenomenon widely observed in nature. The color can be controlled via artificially designed nanostructures, including dielectric diffraction gratings and dielectric photonic crystals [1, 2], dielectric Mie resonance [3, 4], and plasmonic resonance [5], [6], [7], [8], [9], [10], [11], [12], [13]. Wherein plasmonic colors are generated via a resonant interaction between light and metallic nanostructures with subwavelength resolution, colors can be obtained by controlling the geometries and dimensions of the nanostructures [14], [15], [16]. Because of the near-field light localization in these plasmonic nanosystems, plasmonic colors can even be printed with a resolution that exceeds the diffraction limit, indicating that more color information can be encoded in one element [10]. Reflective displays (so-called electronic paper) are one of the important applications in this field [13]. However, producing plasmonic colors that withstand ultrahigh temperatures without damage remains a great challenge. In principle, the shapes of plasmonic nanostructures containing noble metals would change after the heat treatment that altered the plasmonic resonance [17]. Thus, discovering refractory plasmonic materials that can exhibit plasmonic resonance in the visible range is essential. Among the refractory materials, plasmonic transition metal nitrides (TMNs) have received considerable attention due to their outstanding material properties, including low cost, exceptional mechanical hardness, high melting point, and high compatibility with complementary metal–oxide–semiconductor. The unique properties of refractory TMNs show great potential for use as alternative plasmonic materials to replace noble metals [18–22], which would lead to widespread application in machining, microelectronics, and bioelectronics [18, 23], [24], [25], [26], [27], [28], [29], [30]. A challenge in refractory plasmonic materials is the bulk plasmon frequency is usually at the near-infrared range, making it difficult to generate plasmonic colors in the visible. Recently, it has been reported that using alumina-coated gold nanostructures to demonstrate refractory plasmonics properties without using refractory materials [31]. Still, the alumina-coated gold nanostructures only exhibit localized surface plasmon resonance (LSPR) in the near-infrared region. The rapid decay of plasmons in the refractory plasmonic materials due to the large imaginary part of dielectric permittivity results in high system loss, but this loss mechanism provides a pathway to convert photons into hot carriers that can be extracted for a variety of applications, such as solar cells, photocatalysts, and photovoltaic devices [21, 32], [33], [34]. Unlike titanium nitride (TiN) that has been studied intensively in the fields of nanophotonics [25, 35], [36], [37], [38], [39], [40], photocatalysis [41, 42], and photovoltaics [43], research on HfN plasmonic nanostructures remains largely unexplored. Among the TMNs, hafnium nitride (HfN) with a high melting point (T ∼ 3583 K) [20, 22], which exhibits metallic and optical plasmonic characteristics similar to those of gold. HfN has a relatively high bulk plasmon frequency (*λ*_p_ = 400 nm) and a relatively large magnitude of the real part of the permittivity, which enable intense local electromagnetic field confinement to form LSPR in the visible region. The negative real part of the permittivity of HfN with high bulk plasmon frequency of 3.1 eV that could support LSPR in the visible range, which is the key mechanism of full-color generation from the HfN plasmonic nanostructures. Full-visible plasmonic colors that are stable at ultrahigh temperatures remain a fundamental material challenge for ultracompact integrated circuits, high-power photonic devices, reflective displays, and solar energy harvesting systems.

In this work, we realize full-color plasmonic pixels with subdiffraction resolution through tailoring plasmonic crystals made of HfN nanodisk (HfN ND) arrays supported on an ultrathin HfN back reflector. By controlling the absorption peak wavelength of the LSPR with varied plasmonic nanostructure designs, the reflection color can be tuned in the visible wavelength region. Additionally, we experimentally demonstrate that HfN refractory plasmonic crystals (RPCs) can withstand high-temperature annealing without damage. We anticipate that these plasmonic color technologies with thermal-stable and air-stable features will lead to important emerging applications.

## 2 Experimental section

### 2.1 Sample preparation

To experimentally obtain the designed optical properties, HfN plasmonic crystals were fabricated as follows using electron beam lithography. To pattern a nanodisk array, e-beam lithography (FEI) with a fine-tuned e-beam dosage (500–600 μC/cm^2^) and development in a solution with methyl isobutyl ketone (MIBK):IPA. A hard mask of Cr with a thickness of 80 nm was deposited using e-beam evaporation (AST E-Beam Evaporator) and lifted off to remove unnecessary metal. The sputtered HfN film was then selectively etched by plasma gases in a reactive ion etching chamber using chlorine (Ar/Cl_2_ = 4/12 sccm)-based dry etching (Oxford ICP-RIE). Then, the top Cr mask was removed. The etching depth (∼100 nm) was confirmed by a Dektak XT surface profilometer.

### 2.2 Optical measurements

Reflection spectra were obtained on a homemade microspectrophotometer, which consisted of a microscope, a white light source (tungsten-halogen), and a spectrometer. A 20× objective lens (0.4 NA; Olympus) was utilized to ensure near-normal incidence. A circular area of interest with a diameter of 30 µm was selected by the field aperture and confirmed by a CCD camera. The tungsten-halogen white light source was used for illumination. The reflected or transmitted output signal was collected by the spectrometer, with the spectrum covering the spectral range from 400 nm to 900 nm. Specifically, for reflection measurement, the input light and output signal were illuminated or collected from the top of the devices. A reference signal measured on a silver film (40 nm) on a MgO substrate was used to normalize the reflection spectra. All of the optical measurements were carried out in ambient atmosphere at room temperature.

### 2.3 Numerical simulations

The calculated reflection spectrum and electromagnetic field distribution were calculated with the FDTD method (FDTD Solution, Lumerical). The dielectric permittivities of HfN films were extracted from ellipsometry characterization with Drude–Lorentz model fitting. In the case of normal incidence, a broadband plane wave impinged on a single unit cell of the HfN nanodisk array with periodic boundary conditions in the in-plane (*x*–*y*) directions and perfectly matched layer (PML) boundary conditions in the excitation (*z*) direction. A nonuniform mesh with a minimum mesh size of 2 nm covered the whole nanodisk. The results were obtained after the simulation had converged.

## 3 Results and discussion

**Figure 1:**
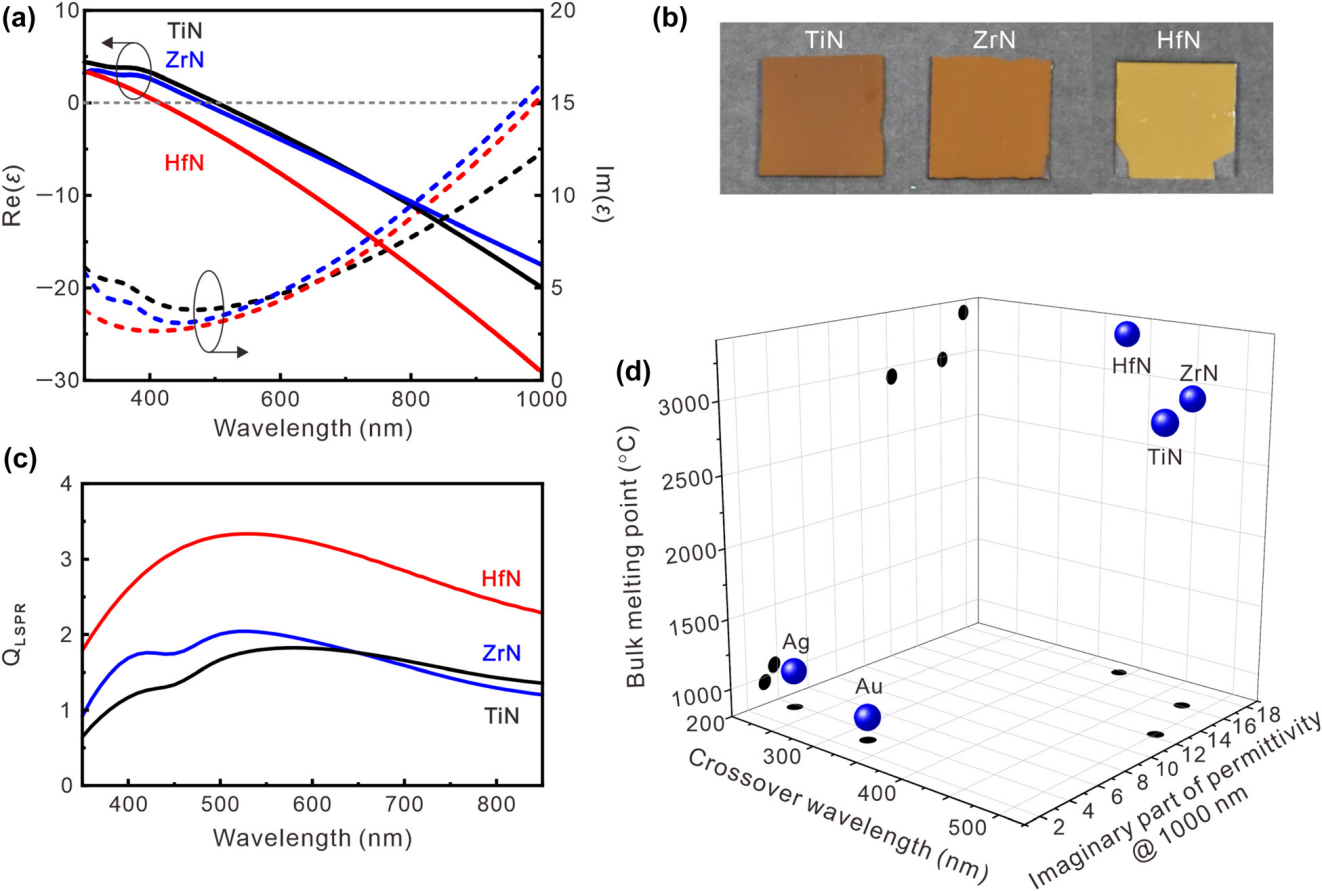
Material characterization of plasmonic transition metal nitride films. (a) Real part (solid line) and imaginary part (dashed line) of the dielectric permittivity of the fabricated plasmonic TiN (black), ZrN (blue), and HfN (red) films. (b) Image of TiN, ZrN, and HfN films on sapphire substrates deposited by RF reactive magnetron sputtering in an ultrahigh-vacuum chamber at 800 °C. (c) Calculated quality factor of the LSPR from the TiN, ZrN, and HfN films. The relatively high quality factor of the LSPR in the HfN films can cover the entire visible spectrum. (d) TiN, ZrN, and HfN provide a relatively high-quality plasmonic resonance in the visible region and refractory properties that can survive under harsh operational conditions.

Distinct from other plasmonic colors using silver, gold, or aluminum nanostructures, we employed an atomically smooth plasmonic HfN film as a refractory plasmonic material. Important for high-power devices, HfN is a durable high-temperature material whose melting point is three times higher than that of gold or other noble plasmonic materials, and its plasmonic properties can be tailored by the growth conditions similar to TiN [36]. In this work, plasmonic HfN, TiN, and ZrN films were sputter-deposited onto sapphire (0001) substrates using radio-frequency (RF) magnetron sputtering at 800 °C (see Tables S1 and S2). The complex optical permittivities of TiN, HfN, and ZrN thin films characterized by a variable angle ellipsometer at angles of 65°–75° are shown in Figure 1(a). Among these three films, the HfN film shows relatively large negative magnitude of the real part of the permittivity, leading to better localized electromagnetic field confinement capability in the visible-NIR spectral range. Figure 1(b) shows an image of the TMN films, and the reflection color of the HfN film is similar to that of gold. To further understand the feasibility for plasmonic applications, we examine the quality factor *Q*_LSPR_ of the LSPR, which is a metric to evaluate the overall performance of plasmonic materials. It is defined as [44],

$$Q_{\text{LSPR}}=\frac{1}{2}\frac{\omega }{\text{lm}\left(\epsilon \right)}\frac{\text{d}}{\text{d}\omega }\text{Re}\left(\epsilon \right)$$

As shown in Figure 1(c), the calculated *Q*_LSPR_ versus the wavelength *λ* reflects that HfN has the best plasmonic performance in terms of plasmonic resonance due to the large negative value of RE(*ε*) with *λ*. However, the increased *Q*_LSPR_ also means a smaller bandwidth and a stronger magnitude of localized field confinement, a general trade-off that needs to be considered. Figure 1(d) shows the summarized results of the material characterization, including silver (Ag), gold (Au), ZrN, TiN, and HfN. Generally, it is known that the transition metal nitrides have much higher melting points than Ag and Au. However, the imaginary part of permittivity at a wavelength of 1000 nm is on the same order of magnitude. Indeed, TiN and ZrN present poor potential for visible wavelength application due to the low bulk plasmon frequency (long crossover wavelength of approximately 480 nm for ZrN and 505 nm for TiN). The HfN film that is characterized by a relatively high-quality factor (Figure 1(c)) in the visible region and a high bulk plasmon frequency (short crossover wavelength of approximately 400 nm) can be utilized for full-color plasmonic pixels due to the ability to generate LSPR in the visible region. In addition, the atomic force microscopy (AFM) image (see Figure S1) reveals that HfN film has an atomically smooth surface which is very important for plasmonic resonance nanostructure design to reduce the scattering loss. Through the control of the absorption peak wavelengths of the LSPR, the reflection color can be tuned in the visible wavelength region.

**Figure 2:**
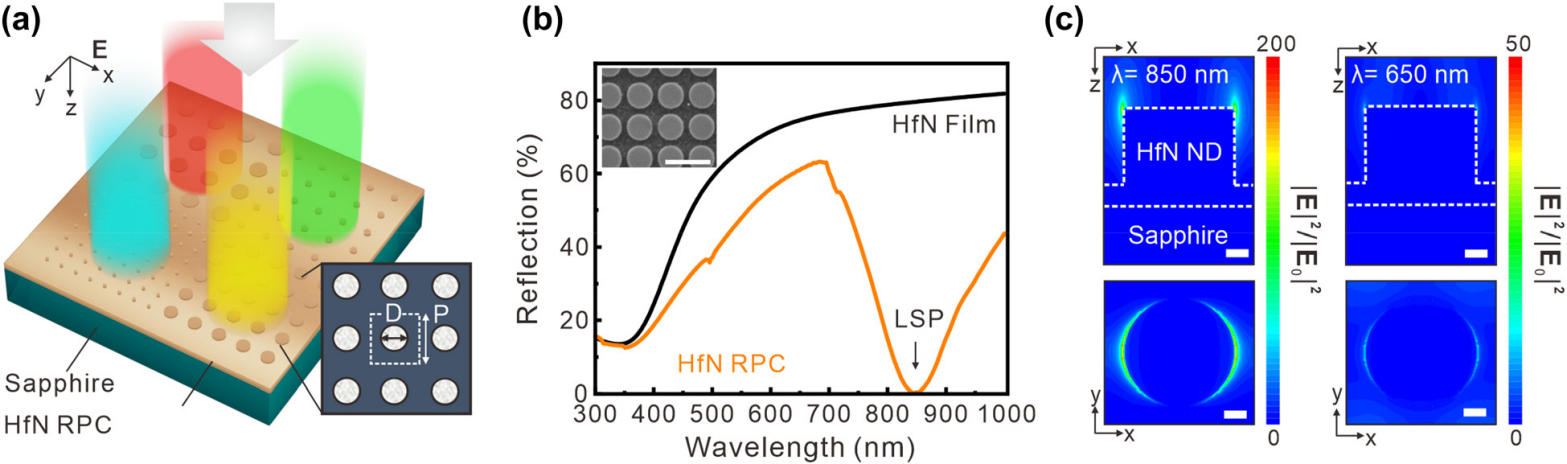
Plasmonic colors using a HfN refractory plasmonic crystals (RPCs). (a) Schematic of the plasmonic crystals that contain HfN nanodisk arrays on a 30 nm HfN back reflector. (b) Measured reflection spectra from the HfN RPC (orange) and HfN film (black). The resonance dip in the reflection spectra at 850 nm originates from the LSPR. The inset shows an SEM image of the HfN RPC. The unit cell of the HfN ND has dimensions of pitch, diameter, and height of *P* = 700 nm, *D* = 520 nm, and *h* = 100 nm. (c) Simulated electric field intensity enhancement distributions in the *x–z* plane and *x–y* plane at *λ* = 850 nm (on resonance) and 650 nm (off resonance).

To demonstrate the visible color performances, we started by designing the LSPR and absorption of HfN plasmonic nanostructures. The proposed HfN RPCs are composed of arrays of HfN nanodisks with various dimensions on an ultrathin HfN back reflector, producing tunable visible color reflections (Figure 2(a)). The scanning electron microscopy (SEM) image shows the designed HfN RPC device, wherein HfN nanodisks with a thickness of 100 nm are arranged in a square lattice with varied pitch (P) and diameter (D). Figure 2(b) shows the comparison of the calculated reflection spectra of the HfN film and HfN RPC. Notably, the designed HfN RPC (D, P) = (520 nm, 700 nm) presents a resonance dip in the reflection spectrum at 850 nm, which is attributed to the LSPR. We further calculated the reflection of a HfN RPC under s- and p-polarized illuminations with a range of incidence angle *θ* from 0° to 60° (see Figure S2), Overall, for the light illumination in both polarizations with varied incident angles, the results show the HfN RPC has an angle-sensitive reflection. To understand the reflection dip originating from the LSPR, we visualized the electric field distributions over the unit cells for the reflection peak and reflection dip of HfN RPCs by finite-difference time-domain (FDTD) simulation, as shown in Figure 2(c). The field profiles show that the localized electromagnetic field is excited only on the top corner of the HfN ND. The electric field exhibits a dipole-like distribution, which is generated from the linearly polarized incident light. Under resonance conditions, the maximum value of the electric field intensity enhancement is approximately 200 at a wavelength of 850 nm, leading to an intense LSPR. In contrast, the maximum value of the electric field intensity dramatically decreases to 50 at a wavelength of 650 nm (off resonance). Furthermore, the reflection spectrum can be tuned by the thin HfN back reflector (approximately 30 nm thickness), which induces a spectral blueshift behavior for the reflection color. This can be interpreted by introducing the reflection phase from the HfN metasurfaces.

**Figure 3:**
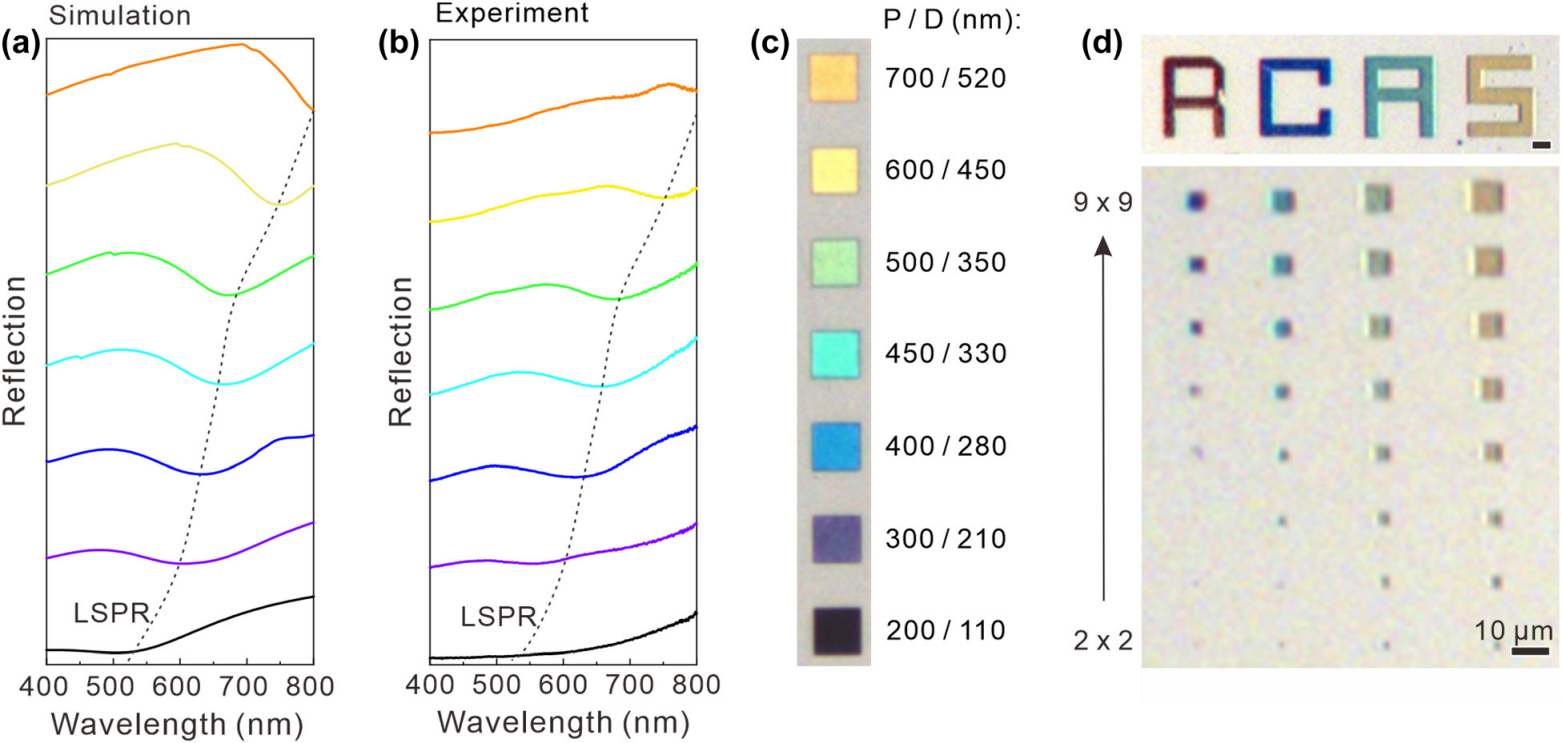
Full-visible colors generated from the HfN refractory plasmonic crystals (RPCs). (a) Simulated and (b) measured reflection spectra from HfN RPCs with various geometries. The resonance dip, originating from LSPR, in the reflection spectrum shifts toward longer wavelengths with both increased diameter (D) and pitch (P) of the HfN nanodisk. (c) Optical microscope images of each plasmonic crystals with an area of 13 μm × 13 μm corresponding to various combinations of *D* and *P* of HfN nanodisk arrays. The reflection colors cover the entire visible spectrum. (d) Optical image of the characters “RCAS” with varied colors. The bottom image reveals the minimum color pixel unit.

A white light was coupled to the HfN plasmonic crystals to further investigate the reflection properties of the fabricated plasmonic nanostructures. The experimental reflection spectra for various diameters and pitches in HfN ND were quantitatively compared to the corresponding spectra simulated using the FDTD method. By tailoring the structural geometry to control the LSPR in the visible region, a wide reflection color range is presented in Figure 3. Figure 3(a)–(b) shows the variation in the reflection spectra from HfN plasmonic crystals with various diameters and pitches. In the simulation, we assumed that the electric field (*x*-axis) of the incident plane wave propagates along the *z*-axis. The complex dielectric permittivity of HfN was measured by ellipsometry (Figure 1(a)), and the refractive index of sapphire was fixed at 1.78. In Figure 3(a), the resonance dip in the reflection spectrum shifts toward longer wavelengths as the diameter and pitch increase. The diameters of the HfN ND with a thickness of 100 nm were varied from 100 to 520 nm, and the disks were arranged in a square lattice with varied pitch from 200 to 700 nm. The experimental reflection color range can be tuned across all visible wavelengths and is in good agreement with the calculation results, i.e. a spectral redshift is observed in the reflection dip position, as shown in Figure 3(b). By tuning the physical geometry of the HfN ND arrays, the LSPR wavelength is shifted, resulting in changes in the optical response. The slight deviation between simulation and measurement results is attributed to the imperfect of the fabricated structure such as round corner of fabricated nanodisks. Figure 3(c) shows a bright-field optical image of the fabricated HfN plasmonic crystals with various geometries of the HfN ND arrays under white light illumination through a 10× objective connected to a charge-coupled device (CCD) camera and a spectroscope. The color phase tends to change from blue to orange with increasing diameter and pitch. The CIE *x–y* chromaticity diagram (see Figure S3) shows the color range of plasmonic colors in Figure 3(c). The calculated results show the reflection color strongly depends on the physical geometry of the nanodisk arrays and the thickness of the back reflector (see Figure S4). To improve the reflection intensity of the designed color, a HfN back reflector is important and both the size of the nanodisk and the pitch are critical. We observed the wavelength of the LSPR as a function of the diameter and the pitch of HfN ND arrays (see Figure S5). This results indicate that a significant color change can be controlled by varying the pitch of the nanodisk arrays. Additionally, color saturation without a large color change can be achieved by varying the diameter of the nanodisk arrays. Figure 3(d) shows the optical image of the characters “RCAS”, with a scale bar of 10 μm. The bottom image reveals the minimum color pixel unit is two-by-two array of nanodisks.

**Figure 4:**
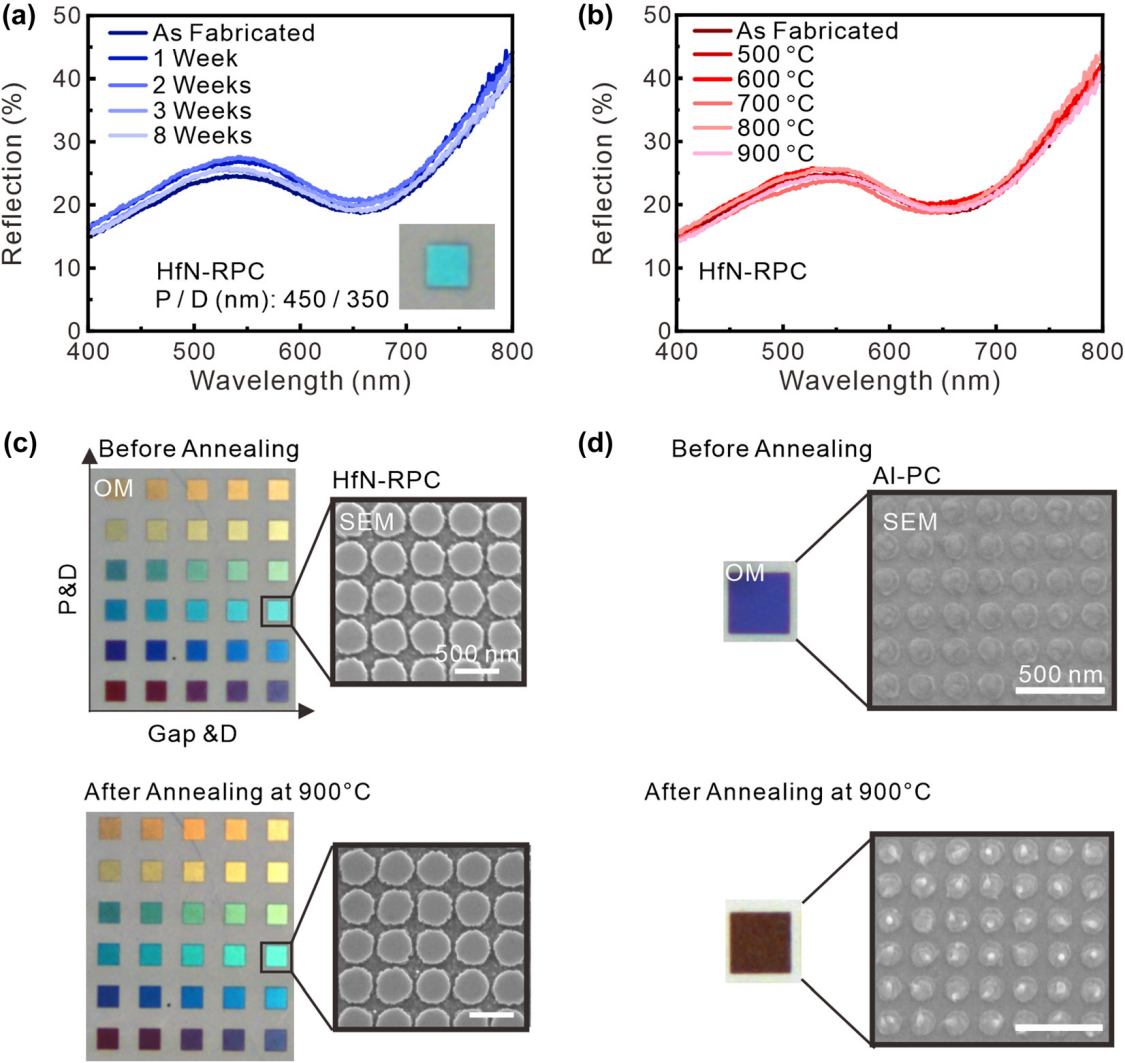
Refractory properties and stability of HfN refractory plasmonic crystals (RPC). (a) The measured reflection spectrum of the HfN RPC remains the same as a function of time, implying no sample degradation under ambient conditions after two months. (b) Measured reflection spectra of the HfN RPC after heating at 500 °C–900 °C for 40 min, revealing no significant variation versus the reference spectrum measured before annealing. (c) Optical images and SEM images of plasmonic HfN RPC and corresponding SEM images before annealing and after 900 °C annealings. (d) Optical images and SEM images of Al-based plasmonic crystals (PC) as a counterpart before and after annealing at 900 °C. The reflection color and the shape of the nanodisks change significantly after the heat treatment.

For ideal color pixels, subdiffraction resolution with environmentally and thermally stable properties is highly desired so that designed plasmonic color pixels are consistent in harsh environments or integrated ultracompact optoelectronic devices. In general, as the device size becomes small, local heat accumulation may damage the plasmonic resonant nanostructures. We found that HfN RPCs show excellent thermal and environmental stabilities. Figure 4(a) shows the measurement results of the reflection spectrum from a specific HfN RPC for different exposure times under standard ambient pressure and temperature for two months, and the reflection spectrum was measured each week. The weekly measured reflection spectra present good air-stable feature, and no spectral shifting is observed. Because of nitrogen atoms bonding with hafnium, oxygen has difficulty oxidizing the HfN surface. Even after two months, the reflection spectrum from the designed HfN RPC remains the same as the originally measured spectrum. To investigate the thermal stability of HfN RPCs, we annealed the devices in a vacuum chamber at 500, 600, 700, 800, and 900 °C for 40 min. Figure 4(b) shows the measured reflection spectra from the HfN RPC with various heating temperatures from 500 to 900 °C. The refection spectra from the heated samples remains the same as that from the fabricated HfN RPC. As indicated by previous investigations, the refractory properties and metallicity of TMNs can be maintained up to above 1000 °C with only a slight degradation in the geometry and optical properties [40, 45], [46], [47]. Due to the high melting point of HfN, the HfN nanodisk arrays change in neither shape nor reflection at 900 °C, as shown in Figure 4(c). The optical images and SEM images show respectively that the full-color pixels display (see Table S3 for the geometry) identical reflection color and the same shape after annealing at 900 °C in a vacuum chamber for 40 min compared with the as-fabricated HfN RPCs. This means that the HfN-based RPCs has outstanding thermal stability. For comparison, noble metal Al-based plasmonic crystals (PCs) with the same metasurface structures (*D* = 410 nm, *P* = 450 nm) were treated in the same way. Although the shapes of plasmonic nanostructures containing noble metals would change after heat treatment, resulting in a significant peak shift of plasmonic resonance [17]. The reason for using an Al-based PCs is that it is a promising candidate for realizing full-color plasmonic pixels with subwavelength resolution [7]. As shown in Figure 4(d), the Al-based PCs heated at 900 °C were melted into different shapes with droplets on the surface, as indicated by the SEM image. The reflection color and spectrum changed significantly (see Figure S6). This results can be interpreted by considering that the melting point of bulk HfN (3385 °C) is much higher than that of Al (660 °C). The measurement results show that the HfN-based RPCs covered the entire visible region with high thermal stability, in contrast to the performance of the Al-based PCs. In addition, we annealed the HfN RPCs by the rapid thermal annealing (RTA) method at 900 °C for 10 min in ambient air for the basic oxidation test (see Figure S7). We observed a significant color change in HfN RPCs, but the shape of the nanodisks remained the same after the RTA treatment. We conclude this is due to the harsh-condition-induced material oxidation. In principle, capping an alumina layer on top of plasmonic nanostructures as a passivation layer could prevent this oxidation issue when operated in a harsh environment [31]. The combination of a refractory nature and the ability to exhibit LSPR in the visible region makes HfN RPCs a promising candidate for robust optoelectronic applications (nonlinear optics, and high-power devices). Since color printing with a subwavelength resolution can be realized by using the proposed nanostructure, we could further design nonsymmetric structure arrays or gate tunable structure arrays that enable information encoding. To further increase the color purity, exploring a new refractory material with lower optical losses in the visible region to obtain high-*Q* resonance is important. In this work, we show that the minimum pixel unit is a two-by-two array of nanodisks (Figure 3(d)), in which RPCs were demonstrated at the optical diffraction limit (400 nm by 400 nm), which leads to achieving a high image resolution of ∼63,500 dpi [12, 48].

## 4 Conclusions

In summary, we have designed and experimentally demonstrated HfN RPCs with a subwavelength resolution. The fabricated HfN RPCs exhibited full-visible colors, which were strongly dependent on the physical geometry of the nanostructure. In contrast to noble metal plasmonic colors, the HfN refractory plasmonic colors enable operation under harsh operational conditions with high image resolution of ∼63,500 dpi. The unique features in HfN, a high bulk plasmon frequency of 3.1 eV (*λ*_p_ = 400 nm), results in full-color plasmonic pixels that can display visible reflective colors. By tuning the wavelength of the LSPR from 520 nm to 850 nm, the reflective optical response can be controlled from blue to red on the subwavelength scale. We verified that the HfN RPCs could work at ultrahigh temperatures without material degradation. We believe that this approach has tremendous potential application in refractory plasmonic color technologies such as in high-power photonic devices, ultracompact integrated circuits, reflective displays, and solar-energy harvesting systems.

## Supplementary Material

Supplementary Material

## References

[1] Arsenault A. C., Puzzo D. P., Manners I., Ozin G. A. (2007). Photonic-crystal full-colour displays. *Nat. Photonics*.

[2] Teyssier J., Saenko S. V., van der Marel D., Milinkovitch M. C. (2015). Photonic crystals cause active colour change in chameleons. *Nat. Commun.*.

[3] Nagasaki Y., Suzuki M., Takahara J. (2017). All-dielectric dual-color pixel with subwavelength resolution. *Nano Lett.*.

[4] Yang J.-H., Babicheva V. E., Yu M.-W., Lu T.-C., Lin T.-R., Chen K.-P. (2020). Structural colors enabled by lattice resonance on silicon nitride metasurfaces. *ACS Nano*.

[5] Ameling R., Dregely D., Giessen H. (2011). Strong coupling of localized and surface plasmons to microcavity modes. *Opt. Lett.*.

[6] Esfandyarpour M., Garnett E. C., Cui Y., McGehee M. D., Brongersma M. L. (2014). Metamaterial mirrors in optoelectronic devices. *Nat. Nanotechnol.*.

[7] Olson J., Manjavacas A., Liu L. (2014). Vivid, full-color aluminum plasmonic pixels. *Proc. Natl. Acad. Sci. U. S. A.*.

[8] Gu Y., Zhang L., Yang J. K. W., Yeo S. P., Qiu C.-W. (2015). Color generation *via* subwavelength plasmonic nanostructures. *Nanoscale*.

[9] Shaltout A. M., Kim J., Boltasseva A., Shalaev V. M., Kildishev A. V. (2018). Ultrathin and multicolour optical cavities with embedded metasurfaces. *Nat. Commun.*.

[10] Neubrech F., Duan X., Liu N. (2020). Dynamic plasmonic color generation enabled by functional materials. *Sci. Adv.*.

[11] Hu H., Gao W., Zang R. (2020). Direct growth of vertically orientated nanocavity arrays for plasmonic color generation. *Adv. Funct. Mater.*.

[12] Joo W.-J., Kyoung J., Esfandyarpour M. (2020). Metasurface-driven OLED displays beyond 10,000 pixels per inch. *Science*.

[13] Xiong K., Olsson O., Svirelis J., Palasingh C., Baumberg J., Dahlin A. (2021). Video speed switching of plasmonic structural colors with high contrast and superior lifetime. *Adv. Mater.*.

[14] Kristensen A., Yang J. K. W., Bozhevolnyi S. I. (2016). Plasmonic colour generation. *Nat. Rev. Mater.*.

[15] Song M., Wang D., Peana S. (2019). Colors with plasmonic nanostructures: a full-spectrum review. *Appl. Phys. Rev.*.

[16] Franklin D., He Z., Ortega P. M. (2020). Self-assembled plasmonics for angle-independent structural color displays with actively addressed black states. *Proc. Natl. Acad. Sci. U. S. A.*.

[17] Chen K.-P., Drachev V. P., Borneman J. D., Kildishev A. V., Shalaev V. M. (2010). Drude relaxation rate in grained gold nanoantennas. *Nano Lett.*.

[18] Naik G. V., Shalaev V. M., Boltasseva A. (2013). Alternative plasmonic materials: beyond gold and silver. *Adv. Mater.*.

[19] Li W., Guler U., Kinsey N. (2014). Refractory plasmonics with titanium nitride: broadband metamaterial absorber. *Adv. Mater.*.

[20] Haynes W. M., Lide D. R., Bruno T. J. (2017). *CRC Handbook of Chemistry and Physics*.

[21] Habib A., Florio F., Sundararaman R. (2018). Hot carrier dynamics in plasmonic transition metal nitrides. *J. Opt.*.

[22] Askes S. H. C., Schilder N. J., Zoethout E., Polman A., Garnett E. C. (2019). Tunable plasmonic HfN nanoparticles and arrays. *Nanoscale*.

[23] Saha B., Acharya J., Sands T. D., Waghmare U. V. (2010). Electronic structure, phonons, and thermal properties of ScN, ZrN, and HfN: a first-principles study. *J. Appl. Phys.*.

[24] Birkholz M., Ehwald K.-E., Wolansky D. (2010). Corrosion-resistant metal layers from a CMOS process for bioelectronic applications. *Surf. Coating. Technol.*.

[25] West P. R., Ishii S., Naik G. V., Emani N. K., Shalaev V. M., Boltasseva A. (2010). Searching for better plasmonic materials. *Laser Photon. Rev.*.

[26] Boltasseva A., Atwater H. A. (2011). Low-loss plasmonic metamaterials. *Science*.

[27] Naik G. V., Kim J., Boltasseva A. (2011). Oxides and nitrides as alternative plasmonic materials in the optical range [Invited]. *Opt. Mater. Express*.

[28] Guler U., Boltasseva A., Shalaev V. M. (2014). Refractory plasmonics. *Science*.

[29] Wang Y., Capretti A., Dal Negro L. (2015). Wide tuning of the optical and structural properties of alternative plasmonic materials. *Opt. Mater. Express*.

[30] Xia H., Wen X., Feng Y. (2016). Hot carrier dynamics in HfN and ZrN measured by transient absorption spectroscopy. *Sol. Energy Mater. Sol. Cell.*.

[31] Albrecht G., Kaiser S., Giessen H., Hentschel M. (2017). Refractory plasmonics without refractory materials. *Nano Lett.*.

[32] Clavero C. (2014). Plasmon-induced hot-electron generation at nanoparticle/metal-oxide interfaces for photovoltaic and photocatalytic devices. *Nat. Photonics*.

[33] Brongersma M. L., Halas N. J., Nordlander P. (2015). Plasmon-induced hot carrier science and technology. *Nat. Nanotechnol.*.

[34] Zhou L., Swearer D. F., Zhang C. (2018). Quantifying hot carrier and thermal contributions in plasmonic photocatalysis. *Science*.

[35] Gui L., Bagheri S., Strohfeldt N. (2016). Nonlinear refractory plasmonics with titanium nitride nanoantennas. *Nano Lett.*.

[36] Lu Y. J., Sokhoyan R., Cheng W. H. (2017). Dynamically controlled Purcell enhancement of visible spontaneous emission in a gated plasmonic heterostructure. *Nat. Commun.*.

[37] Minn K., Anopchenko A., Chang C.-W. (2021). Enhanced spontaneous emission of monolayer MoS_2_ on epitaxially grown titanium nitride epsilon-near-zero thin films. *Nano Lett.*.

[38] Mishra R., Chang C.-W., Dubey A. (2021). Optimized titanium nitride epitaxial film for refractory plasmonics and solar energy harvesting. *J. Phys. Chem. C*.

[39] Lu Y.-J., Shen T. L., Peng K.-N. (2021). Upconversion plasmonic lasing from an organolead trihalide perovskite nanocrystal with low threshold. *ACS Photonics*.

[40] Fomra D., Mamun M., Ding K., Avrutin V., Özgür Ü., Kinsey N. (2021). Plasmonic colors in titanium nitride for robust and covert security features. *Opt Express*.

[41] Naldoni A., Guler U., Wang Z. (2017). Broadband hot-electron collection for solar water splitting with plasmonic titanium nitride. *Adv. Opt. Mater.*.

[42] Yu M.-J., Chang C.-L., Lan H.-Y. (2021). Plasmon-enhanced solar-driven hydrogen evolution using titanium nitride metasurface broadband absorbers. *ACS Photonics*.

[43] Wang H., Chen Q., Wen L., Song S., Hu X., Xu G. (2015). Titanium-nitride-based integrated plasmonic absorber/emitter for solar thermophotovoltaic application. *Photon. Res.*.

[44] Wang F., Shen Y. R. (2006). General properties of local plasmons in metal nanostructures. *Phys. Rev. Lett.*.

[45] Naik G. V., Saha B., Liu J. (2014). Epitaxial superlattices with titanium nitride as a plasmonic component for optical hyperbolic metamaterials. *Proc. Natl. Acad. Sci. U. S. A.*.

[46] Briggs J. A., Naik G. V., Zhao Y. (2017). Temperature-dependent optical properties of titanium nitride. *Appl. Phys. Lett.*.

[47] Reddy H., Guler U., Kudyshev Z., Kildishev A. V., Shalaev V. M., Boltasseva A. (2017). Temperature-dependent optical properties of plasmonic titanium nitride thin films. *ACS Photonics*.

[48] Kumar K., Duan H., Hegde R. S., Koh S. C. W., Wei J. N., Yang J. K. W. (2012). Printing colour at the optical diffraction limit. *Nat. Nanotechnol.*.

