# Supplementary material for: Full-color generation enabled by refractory plasmonic crystals
Source: Nanophotonics. 2022 May 6;11(12):2891–9. doi: 10.1515/nanoph-2022-0071 (PMC11501569; doi:10.1515/nanoph-2022-0071)
Supplement: Supplementary file 1 — Supplementary Material [file j_nanoph-2022-0071_suppl.pdf]

Supplementary Information for

Full-Color Generation Enabled by Refractory Plasmonic Crystals

Zong-Yi Chiao,[1#] Yu-Chia Chen,[1#] Jia-Wern Chen,[1] Yu-Cheng Chu,[1,2] Jing-Wei Yang,[1,2] Tzu-Yu Peng,[1,2] Wei-Ren Syong,[1] Ho Wai Howard Lee,[3] Shi-Wei Chu,[2,4,5] and Yu-Jung Lu[1,2*]

[1]*Research Center for Applied Sciences, Academia Sinica, Taipei 11529, Taiwan*
[2]*Department of Physics, National Taiwan University, Taipei 10617, Taiwan*
[3]*Department of Physics & Astronomy, University of California, Irvine, CA 92697, United States*
[4]*Molecular Imaging Center, National Taiwan University, Taipei 10617, Taiwan*
[5]*Department of Physics, National Tsing Hua University, Hsinchu 30013, Taiwan*

[#] Zong-Yi Chiao and Yu-Chia Chen contributed equally to this work.

*To whom correspondence should be addressed.

*E-mail : yujunglu@gate.sinica.edu.tw

This PDF file includes:

Note S1. Characterization of plasmonic transiton metal nitride films

Note S2. Atomically smooth HfN film

Note S3. Numerically calculated angle-dependent reflection spectra of the HfN plasmonic crystals

Note S4. CIE *x-y* chromaticity diagram

Note S5. Reflection color depends on the thickness of the HfN thin film

Note S6. Full colors generated from HfN refractory plasmonic crystals with various geometries

Note S7. Thermal stability of plasmonic crystals

Note S1. Characterization of plasmonic transiton metal nitride films

Sample	HfN	TiN	ZrN
**Target**	HfN (99.9%)	TiN (99.9%)	ZrN (99.9%)
**Base pressure (Torr)**	8×10^{-9}	1×10^{-8}	4.8×10^{-9}
**Growth pressure (mTorr)**	3	3	3
**Ar gas flow (sccm)**	12	12	13
**RF bias (V)**	370	380	398
**Growth temperature (°C)**	800	800	800
**Thickness (nm)**	114.7	129.3	69.3
**Substrate**	Sapphire	MgO	Si

Table S1. Growth parameters of the HfN, TiN, and ZrN films by RF sputtering.

The complex permittivity ε of TiN films can be described by a combination of Drude model and Lorentz model measured by using variable angle spectroscopic ellipsometry (VASE, J.A. Woollam Co.) [1], which account for intraband and interband transitions, respectively:

$$\varepsilon = \mathrm{Re}(\varepsilon) + i\,\mathrm{Im}(\varepsilon) = \varepsilon_\infty - \frac{\omega_p^2}{\omega^2 + i\Gamma\omega} + \sum_{j=1}^{n} \frac{f_j \cdot \omega_{0j}^2}{\omega_{0j}^2 - \omega^2 - i\gamma_j\omega} \qquad \text{Equation(1)}$$

In the above equation, complex permittivity ε is a function of the photon frequency. The first term ε_∞ is the high frequency permittivity. The second term corresponds to the Drude model, which accounts for the interaction between conduction electrons and electromagnetic waves, represents the plasma frequency due to conduction electrons and Γ represents the collision rate. The third term corresponds to Lorentz oscillators, which models the absorption contribution from bound electrons, in which f_j is the strength of the oscillator, ω_{oj} is the resonant angular frequency, and γ_j is the damping factor.

	High frequency permittivty	Drude term (intraband)		Lorentz oscillator 1 (interband)			Lorentz oscillator 2 (interband)			ENZ frequency	Fitting MSE
**Parameter**	ε_∞	ω_p (eV)	Γ (eV)	f_1	ω_{o1} (eV)	γ_1	f_2	ω_{o2} (eV)	γ_2	ω_s (eV)	MSE
**HfN**	3.64	8.00	0.49	2.86	5.45	2.96	0.43	1.44	0.90	3.18	1.26
**TiN**	3.6	6.88	0.4	2.5	1.4	2.47	7.94	4.49	2.07	2.48	1.73
**ZrN**	3.99	7.27	0.83	0.29	3.40	0.74	1.87	4.49	1.41	2.58	2.11

Table S2. Ellipsometry fitting parameters for the complex permittivity of HfN, TiN, and ZrN films. The Drude-Lorentz model consists of a Drude term and two Lorentz oscillators.

Note S2. Atomically smooth HfN film

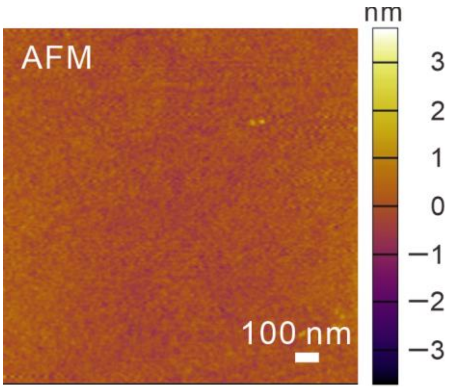

Figure S1. AFM image of an atomically smooth HfN film on sapphire with an RMS of approximately 0.5 nm.

Note S3. Numerically calculated angle-dependent reflection spectra of the HfN plasmonic crystals

The incident angle θ is defined as the intersection between the wave vector k and the normal vector of the HfN RPC. Under s-polarized light illumination (see Figure S2b), the calculated reflection spectra exhibit a resonance dip in the reflection spectrum shifts toward longer wavelengths as increasing θ. With the p-polarized incidence light (see Figure S2c), we also observed the change of the reflection spectrum with the angle of incidence. Overall, for the light illumination in both polarizations with varied incident angles, the results show the HfN plasmonic crystals have an angle-sensitive reflection.

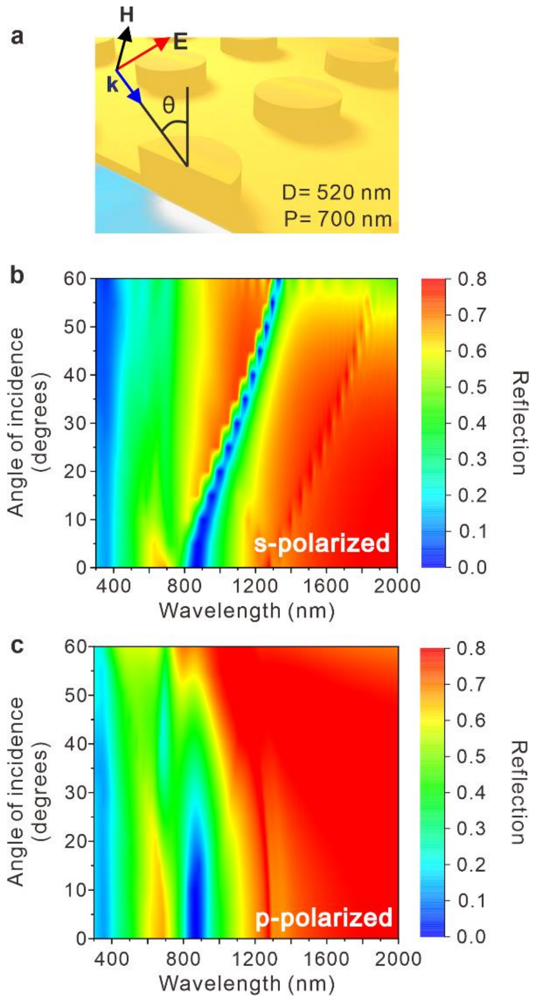

Figure S2. Numerically calculated angle-dependent reflection spectra of HfN plasmonic crystals. (a) Schematic of the HfN plasmonic crystals illuminated by s-polarized light with incident angles varying from 0° to 60°. The unit cell of the HfN nanodisks has dimensions for pitch, diameter, and height of P= 700 nm, D= 520 nm, and h= 100 nm. (b)(c) Color maps for reflection spectra as a function of incident angle under illumination with s-polarized, and p-polarized light.

<u>Note S4. CIE *x-y* chromaticity diagram</u>

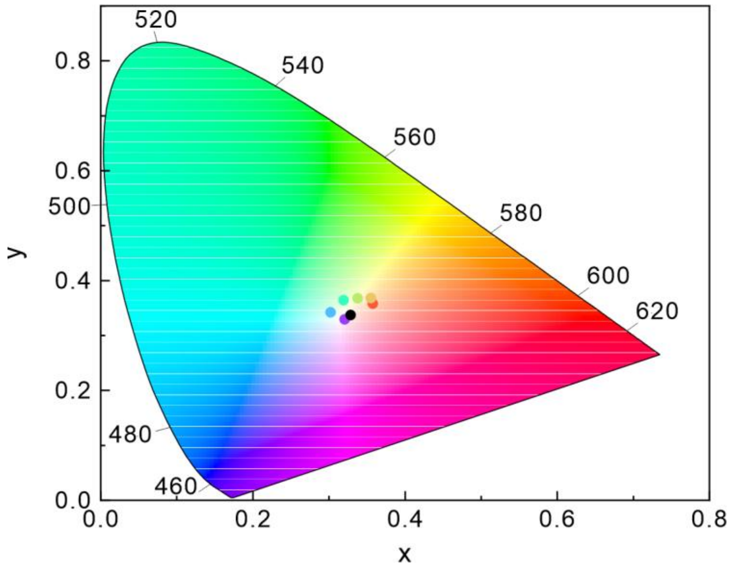

Figure S3. CIE *x-y* chromaticity diagram showing the color range of plasmonic colors in Figure 3c with color plotted in the background of the RGB color space.

Note S5. Reflection color depends on the thickness of the HfN thin film

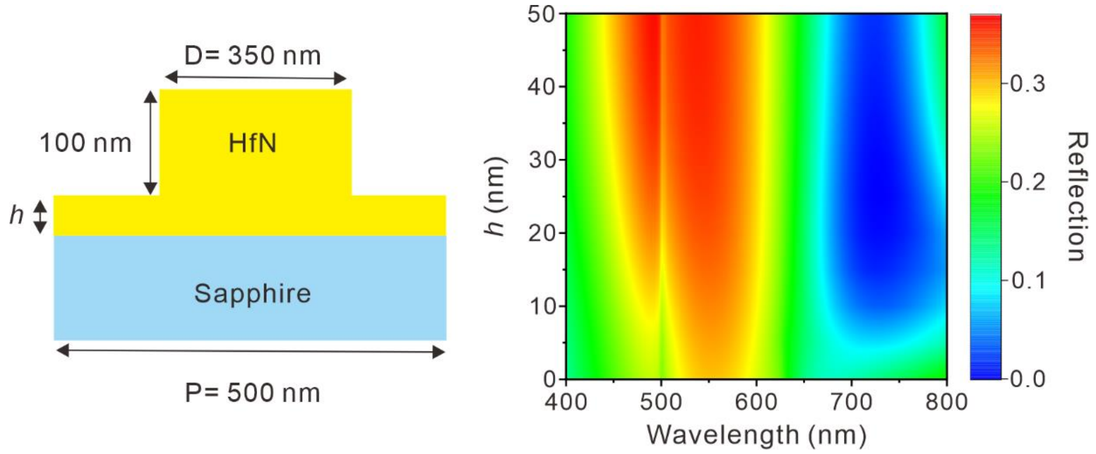

Figure S4. Schematic of the plasmonic pixels that contain HfN nanodisk arrays on a HfN thin film with varied thickness (h). The geometric parameters of the HfN nanodisk arrays are P= 500 nm, D= 350 nm, and height= 100 nm. Color map of numerically calculated reflection spectra as a function of the thickness of the HfN thin film with fixed diameter, pitch, and height of the HfN nanodisk arrays. The plot shows that the thickness of the HfN thin film plays an important role in the reflection color.

Note S6. Full colors generated from HfN refractory plasmonic crystals with various geometries

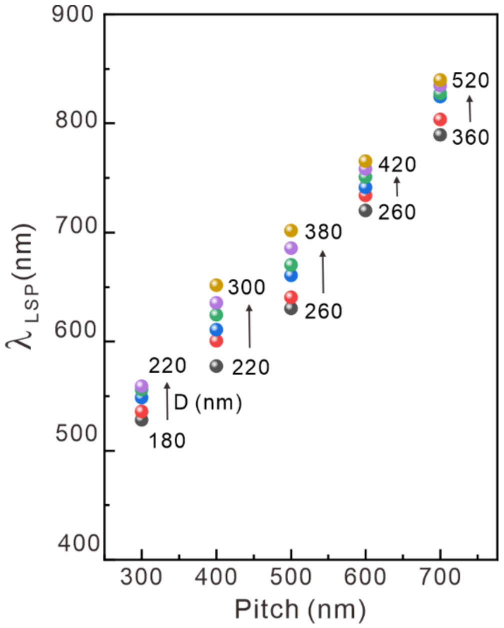

Figure S5. Resonance wavelength of the localized surface plasmon resonance as a function of the diameter and pitch of the HfN nanodisk arrays.

Pitch (nm)	Diameter (nm)				
700	360	400	440	480	520
600	260	300	340	380	420
500	260	290	320	350	380
450	270	290	310	330	350
400	220	240	260	280	300
300	180	190	200	210	220

Table S3. HfN refractory plasmonic crystals with various geometries. Each plasmonic color pixel, as shown in Figure 4, corresponds to various combinations of diameter and pitch of the HfN nanodisk arrays.

Note S7. Thermal stability of plasmonic crystals

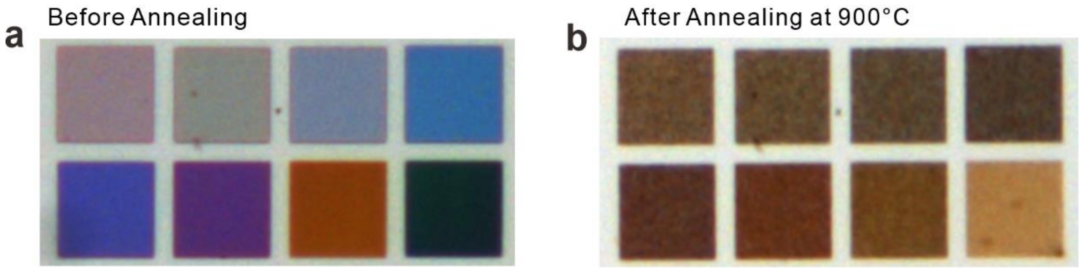

Figure S6. Optical images of Al-based plasmonic crystals before annealing and after 900°C annealing treatment in vacuum. Significant color changes can be observed.

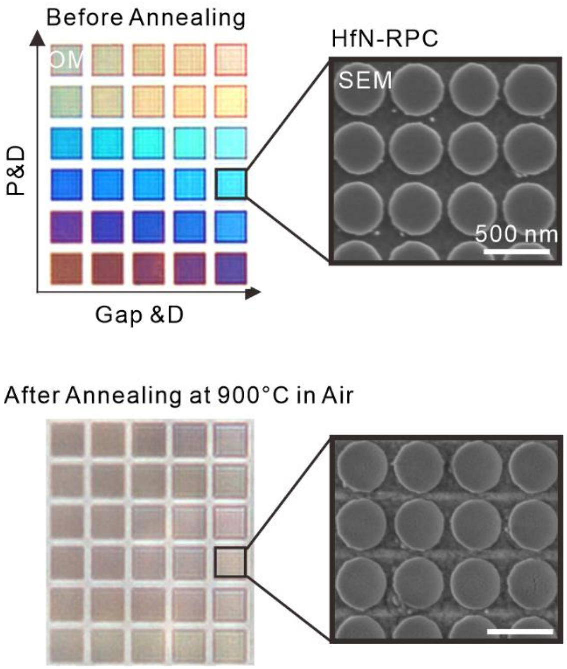

Figure S7. Optical images and SEM images of HfN-based plasmonic crystals before and after annealing at 900°C for 10 minutes in ambient air using the rapid thermal annealing (RTA) method. HfN-based plasmonic crystals show significant changes in the reflection color after heating over 900 °C in ambient air due to the material oxidation.

References

[1]. J. N. Hilfiker, N. Singh, T. Tiwald, D. Convey, S. M. Smith, J. H. Baker, and H. G. Tompkins, "Survey of methods to characterize thin absorbing films with Spectroscopic Ellipsometry," *Thin Solid Films, vol.* 516, pp. 7979–7989, 2008.
